# Minimally Invasive Surgery as a Longitudinal Diagnostic and Restaging Tool in a Recurrent STK11-Altered Adnexal Tumor: A Case Report

**DOI:** 10.3390/diagnostics16101415

**Published:** 2026-05-07

**Authors:** Antonio Maccio, Manuela Neri, Valerio Vallerino, Sonia Nemolato, Gabriele Sole, Elisabetta Pusceddu, Paolo Albino Ferrari

**Affiliations:** 1 Department of Obstetrics and Gynecology and Gybecological Oncology, Azienda di Rilievo Nazionale ed Alta Specializzazione “G. Brotzu”, Piazza A. Ricchi 1, 09121 Cagliari, Italy; antoniopm.maccio@aob.it (A.M.); manuela.neri@aob.it (M.N.); valerio.vallerino@aob.it (V.V.); 2 Department of Oncological Surgery, Azienda di Rilievo Nazionale ed Alta Specializzazione “G. Brotzu”, Piazza A. Ricchi 1, 09121 Cagliari, Italy; gabriele.sole@aob.it; 3 Unit of Anatomic Pathology, Azienda di Rilievo Nazionale ed Alta Specializzazione “G. Brotzu”, Piazza A. Ricchi 1, 09121 Cagliari, Italy; sonia.nemolato@aob.it; 4 Anesthesia and Intensive Care Unit, Liver Transplantation Center, Azienda di Rilievo Nazionale ed Alta Specializzazione “G. Brotzu”, Piazza A. Ricchi 1, 09121 Cagliari, Italy; elisabetta.pusceddu@aob.it

**Keywords:** STK11, LKB1, adnexal tumor, FATWO, molecular reclassification, minimally invasive surgery, CD117, c-KIT, everolimus

## Abstract

**Background and Clinical Significance**: Rare adnexal tumors with Wolffian or sex cord-like differentiation may undergo major diagnostic reclassification after integrated histologic and molecular review. The contribution of minimally invasive surgery to this process has rarely been described in detail. **Case Presentation**: We focused on the case of a 33-year-old woman underwent laparoscopic right salpingectomy in October 2021 for a right-sided tubo-adnexal lesion initially diagnosed as adult granulosa cell tumor and later reinterpreted as high-grade endometrioid carcinoma. Completion staging in February 2022 was negative. Positron emission tomography/computed tomography in January 2023 raised concern for recurrence, and exploratory laparoscopy in March 2023 documented peritoneal metastatic disease, followed by four cycles of cisplatin given within the then-prevailing carcinoma-based diagnostic framework. A second laparoscopic reassessment in October 2023 was negative. Because of multifocal abdominal relapse, the patient underwent major cytoreductive surgery in October 2024. Integrated pathologic and molecular review then documented serine/threonine kinase 11 alteration together with forkhead box L2 negativity, favoring classification within the STK11-altered adnexal tumor spectrum. After external review, everolimus plus anastrozole was started in May 2025. Imaging in late 2025 documented persistent pelvic recurrence, and salvage xipho-pubic laparotomy in December 2025 revealed extensive disease involving both ureters, the bladder base, the rectosigmoid wall, and parietal peritoneum; recurrent tissue showed cluster of differentiation 117 positivity. At the most recent available follow-up in April 2026, the patient had no documented death and remained under postoperative surveillance. **Discussion and Conclusions**: This case illustrates the diagnostic importance of repeated tissue reassessment and the practical value of minimally invasive surgery as a relatively low-burden means of resection, restaging, and tissue acquisition in a rare molecularly reclassified adnexal tumor.

## 1. Introduction

Female adnexal tumor of probable Wolffian origin (FATWO) is a rare neoplasm historically believed to arise from mesonephric duct remnants and first described by Kariminejad and Scully in 1973 [1]. Although many cases follow an indolent course, recurrence and distant spread are well documented, and the long-term biologic behavior of these tumors can be unpredictable [2,3].

During the last several years, the diagnostic boundaries of this group have been refined by molecular pathology. Morphologic, immunohistochemical, and genomic studies have shown that some tumors previously classified as FATWO harbor recurrent serine/threonine kinase 11 (STK11) alterations and likely represent a distinct STK11-associated adnexal neoplasm rather than classical FATWO [4,5,6,7]. This distinction is clinically relevant because disruption of STK11, also known as liver kinase B1 (LKB1), may provide a biologic rationale for mammalian target of rapamycin (mTOR)-directed therapy, whereas KIT proto-oncogene receptor tyrosine kinase (c-KIT) expression has motivated exploratory use of imatinib in selected recurrent cases [8,9,10,11,12,13].

Here, we report a recurrent STK11-altered adnexal tumor with later cluster of differentiation 117 (CD117) positivity by immunohistochemistry, corresponding to c-KIT expression that was managed over several years through repeated minimally invasive procedures, systemic therapy, molecular reclassification, and eventual open salvage surgery. The case is noteworthy less for a single dramatic intervention than for the way serial laparoscopy repeatedly supplied the tissue and staging information required to reinterpret the biology of the disease and redirect treatment.

## 2. Case Presentation

### 2.1. Clinical Course

A 33-year-old woman with no relevant prior medical history underwent laparoscopic right salpingectomy in October 2021 for a right-sided tubo-adnexal lesion. The initial histologic diagnosis was adult granulosa cell tumor. A slide review performed in November 2021 at another tertiary center reclassified the lesion as ovarian endometrioid carcinoma, grade 3. In February 2022, the patient underwent completion surgical staging with pelvic and para-aortic lymphadenectomy; no tumor cells were identified in the staged specimens.

After close clinical and radiologic surveillance, whole-body positron emission tomography/computed tomography (PET/CT) in January 2023 showed a hypermetabolic right common iliac lymph node suspicious for recurrence. Exploratory laparoscopy performed on 8 March 2023 included resection of the left and anterior pelvic peritoneum, right adnexectomy, appendectomy, and omentectomy. Histology demonstrated peritoneal metastatic carcinoma. At that stage, however, the working diagnosis remained recurrent carcinoma rather than a molecularly defined rare adnexal neoplasm, and from May to July 2023 the patient received first-line cisplatin at 75 mg/m^2^ every 21 days for four cycles as an empiric platinum-based approach within that diagnostic framework. Because of subsequent radiologic concern for nodal recurrence, she underwent an additional exploratory laparoscopy with pelvic lymphadenectomy in October 2023; this procedure was negative for tumor. Representative functional imaging from the recurrent phase is provided in Figure 1.

Follow-up PET/CT raised concern for multifocal abdominal recurrence, and in October 2024 the patient underwent further major surgery consisting of total hysterectomy and left adnexectomy, thereby completing adnexal removal after the prior right adnexectomy, together with resection of a bulky right external iliac lymph node and placement of a right external iliac vascular graft. The postoperative course was complicated by right-leg compartment syndrome requiring fasciotomy, followed by gluteal abscess and a left anterior transrectal perianal fistula managed conservatively. Representative pre-operative cross-sectional imaging before salvage laparotomy is shown in Figure 2.

### 2.2. Pathologic and Molecular Reassessment

During the 2024 diagnostic work-up, extended pathologic and molecular reassessment substantially changed the nosologic interpretation. The revised pathology from the October 2024 surgery still described high-grade adult granulosa cell tumor involving the peritoneal specimen and all four examined lymph nodes, and specifically recommended forkhead box L2 (FOXL2) mutational analysis. A subsequent addendum dated 20 January 2025, issued after additional molecular evaluation and external consultation, documented the STK11 variant p.(Leu290Pro), c.869T>C, in exon 7, identified by massively parallel next-generation sequencing (NGS), together with FOXL2 negativity, and favored an STK11-positive adnexal tumor of probable mesonephric/sex cord derivation, described as tubo-ovarian in origin from mesonephric remnants. Variant allele frequency was not retrievable from the available external report. Morphologically, the recurrent tumor showed a nested and tubule-like proliferation embedded in fibrous stroma. That pattern overlapped with prior diagnostic considerations, including FATWO and other sex cord-like or mesonephric/Mullerian mimics, and therefore was not sufficiently specific on its own. In practical terms, the final classification rested on integrated reinterpretation of the recurrent material rather than on morphology alone, with the molecularly supported shift away from granulosa-cell and conventional Mullerian categories providing the decisive argument (Figure 3; Table 1).

On 10 April 2025, the case underwent external oncologic consultation and pathology review at the European Institute of Oncology. After multidisciplinary discussion on 5 May 2025, treatment with everolimus plus anastrozole was recommended and started on 13 May 2025. This choice reflected the absence of a standard systemic regimen for this entity, the biologic rationale for mammalian target of rapamycin (mTOR) inhibition in STK11-altered disease, and the limited published precedent after external expert review. Immunohistochemical testing showed estrogen receptor (ER) positivity and progesterone receptor (PR) positivity in both the primary tumor and the recurrent/re-biopsy specimen. In this context, anastrozole was maintained in combination with everolimus because the tumor displayed a concordant ER+/PR+ profile across disease phases, thereby providing a biologic rationale for endocrine therapy in addition to mTOR inhibition. This treatment choice remained individualized and was also consistent with the limited case-report level experience available in STK11-mutated FATWO-like disease, including the report by Estevez-Diz et al. [8]. The pathology documentation generated during this diagnostic transition also recommended genetic screening for Peutz–Jeghers syndrome, underscoring that the molecular finding had implications beyond histologic relabeling alone.

### 2.3. Imaging Progression and Salvage Surgery

Cross-sectional imaging in late 2025 documented persistent pelvic disease. The clinical record described recurrent pelvic and right iliac fossa lesions with an abscess-like or colliquative appearance on contrast-enhanced computed tomography (CT) reassessment, and magnetic resonance imaging (MRI) confirmed pelvic recurrence contiguous with the vaginal cuff together with multiple bilateral obturator and iliac lymph nodes suspicious for relapse. The images available for publication showed persistent metabolically active pelvic disease on PET/CT dated 29 August 2024 and 4 March 2025, recurrent pelvic soft-tissue disease on CT dated 5 November 2025, and a persistent pelvic lesion on MRI dated 20 November 2025, with one focus measuring approximately 23.7 mm (Figure 1 and Figure 2).

Because of progressive pelvic recurrence, the patient underwent xipho-pubic laparotomy on 1 December 2025. Surgery included resection of a pelvic metastasis involving both ureters, the bladder base, and the rectosigmoid wall; resection of parietal peritoneal metastases; left ureteral anastomosis with double-J stent placement; and closure of the right ureter. On 2 December 2025, a right nephrostomy was placed. Final pathology documented multifocal involvement of the parietal peritoneum, pelvic peritoneum, mesosigmoid adipose tissue, periureteral tissue bilaterally, perivesical peritoneum, and perirectal peritoneum, with bilateral sparing of the ureteral mucosa. Nodal disease burden was substantial, with metastases identified in all five nodes from the right iliac artery/vein packet, all six examined right iliac nodes, and all three right obturator nodes; vascular emboli were present in the obturator nodal/perinodal tissue. The final pathologic impression was multiple localizations of an STK11-altered tubo-ovarian adnexal tumor of probable mesonephric-remnant origin. Immunohistochemistry showed CD117 positivity in approximately 40% of neoplastic cells. At the most recent available follow-up in April 2026, the patient remained under postoperative surveillance, with no documented death (Table 2).

## 3. Discussion

The present case shows how difficult these rare adnexal tumors can be to classify when morphology is interpreted in isolation. Over several years, the same neoplasm was read sequentially as adult granulosa cell tumor, high-grade endometrioid carcinoma, peritoneal metastatic carcinoma, and finally an STK11-altered adnexal tumor of probable mesonephric-remnant origin. Each label was understandable within the information then available, yet each also proved incomplete. In fact, the 2024 revised pathology still favored high-grade adult granulosa cell tumor and documented nodal metastases before recommending FOXL2 testing. The subsequent molecular addendum, which demonstrated STK11 alteration together with FOXL2 negativity, provided the key discordant evidence that shifted the interpretation away from a canonical granulosa-cell neoplasm. Compared with conventional Mullerian carcinoma, the later nested and tubule-like morphology in fibrous stroma together with STK11 alteration and expert external review also argued against a typical epithelial ovarian neoplasm. Compared with classical FATWO, the cumulative profile fit more closely with the recently delineated STK11-altered adnexal spectrum. The diagnostic shift was therefore based on staged contradiction and integration, not on any single microscopic feature [4,5,6,7,14,15].

Recent work by Bennett and colleagues suggests that STK11-altered adnexal tumors represent a distinct clinicopathologic entity, often paratubal and in some patients associated with Peutz-Jeghers syndrome, with a clinical course that may be more aggressive than expected for conventional FATWO [7]. Earlier genomic studies in tumors historically classified as FATWO had already identified recurrent STK11 alterations in a subset of cases, implying that at least part of the older literature probably mixed biologically different lesions under the same label [4,5,6]. In our patient, the pathology addendum did more than simply report an STK11 abnormality: it explicitly favored an STK11-positive adnexal tumor of probable mesonephric/sex cord derivation and described a tubo-ovarian origin from mesonephric remnants, while also recommending genetic screening for Peutz-Jeghers syndrome. Those details make the final classification more defensible and also emphasize that the molecular result had potential hereditary implications. In our patient, the external molecular addendum identified STK11 p.(Leu290Pro), c.869T>C, in exon 7 by massively parallel NGS, together with FOXL2 negativity, thereby making the final classification more specific at the molecular level. At the same time, variant allele frequency and germline STK11 status remained unavailable, which still limits a more granular molecular-pathologic correlation. Key articles that frame the current diagnostic and therapeutic understanding of these tumors are summarized in Table 3.

The relevance of minimally invasive surgery in this case was practical rather than merely technical. In this patient, laparoscopy repeatedly served three concrete purposes: initial low-burden excision, histologic confirmation of recurrence when imaging became suspicious, and restaging when nodal relapse was uncertain. Each procedure yielded information that changed management, either by documenting recurrent disease, excluding it, or providing tissue for later reinterpretation. The case therefore illustrates minimally invasive surgery less as an oncologic endpoint in itself than as a longitudinal diagnostic and strategic tool within a multimodality pathway. Its limits were equally clear: once the recurrence became infiltrative and anatomically complex, radical open surgery was necessary.

Systemic treatment in recurrent FATWO-like and STK11-altered adnexal tumors remains empirical. Platinum-based chemotherapy has been used most frequently, with heterogeneous results, and surgery remains central whenever complete or meaningful cytoreduction is feasible [3,10,13,14]. The earlier use of cisplatin monotherapy in our patient should be interpreted within the historical diagnostic frame of recurrent carcinoma; at that stage, molecular reclassification had not yet occurred and no regimen specific to an STK11-altered adnexal tumor was available. After STK11 alteration was identified, everolimus plus anastrozole was selected after external review because of the biologic rationale for mTOR pathway inhibition and the precedent described by Estevez-Diz et al. in a similarly reclassified tumor [8,9]. In the present case, this choice was further supported by concordant ER+/PR+ immunophenotype in both the primary and recurrent/re-biopsy specimens, which provided a concrete rationale for maintaining anastrozole alongside everolimus. Nevertheless, treatment selection still reflected individualized multidisciplinary extrapolation from limited literature rather than a standardized receptor-driven strategy for this rare entity, and subsequent imaging and operative findings still indicated persistent pelvic disease. STK11-informed treatment selection may therefore be biologically rational without being predictably effective.

The demonstration of CD117 positivity adds another clinically relevant layer. C-KIT expression has been reported in recurrent FATWO and has prompted off-label imatinib use in isolated cases [10,11,12,13]. Published experience, however, also shows that immunohistochemical positivity alone does not establish the presence of an activating KIT alteration or predict durable response to tyrosine-kinase inhibition [11,12,13]. In our patient, CD117 immunohistochemistry was positive in both disease assessments. This repeated finding broadened the biologic discussion but could not by itself define a validated target. KIT molecular testing was not pursued mainly for timing reasons, because the relevant therapeutic decisions had to be made within a limited time window in the setting of progressive disease and surgical salvage planning. Accordingly, the diagnostic work-up prioritized immediately available histopathologic and immunohistochemical information rather than additional molecular testing with a longer turnaround time. We therefore interpret repeated CD117 positivity as potentially informative, but not as proof of actionable KIT dependence in the absence of mutational analysis.

Several limitations should be acknowledged. This report is based on retrospective review of the available clinical records, pathology summaries, and selected images. A complete and uniform immunohistochemical panel across all disease phases was not retrievable; detailed operative documentation from every prior procedure was not available for full comparison; most importantly, although the exact STK11 variant and sequencing approach were later recovered (p.(Leu290Pro), c.869T>C in exon 7, by massively parallel NGS), variant allele frequency and broader assay details were not available from the retrievable external report; germline STK11 status was unknown despite explicit recommendation for Peutz-Jeghers syndrome screening; and follow-up after the December 2025 salvage surgery, although extended to April 2026, remains short and oncologically immature for assessing the durability of disease control after a major salvage procedure. Even so, the case remains informative because its central lesson is practical: in rare adnexal tumors with shifting histologic interpretation, serial tissue reassessment may be decisive, and minimally invasive surgery may be an efficient way to obtain it.

## 4. Conclusions

This longitudinal case suggests that the value of minimally invasive surgery in rare adnexal neoplasms may extend well beyond cytoreduction alone. In our patient, serial laparoscopic procedures repeatedly created opportunities to clarify suspected recurrence, obtain new tissue, and support successive diagnostic reinterpretations until the disease could be more convincingly placed within the STK11-altered adnexal tumor spectrum. The case also highlights how morphology in isolation may be insufficient in FATWO-like tumors and how meaningful management decisions may emerge only from the stepwise integration of surgery, pathology, molecular testing, and expert review over time. When disease remains anatomically amenable to repeated minimally invasive reassessment, this strategy may offer substantial diagnostic and restaging value, even if definitive open salvage surgery ultimately becomes necessary.

## Figures and Tables

**Figure 1:**
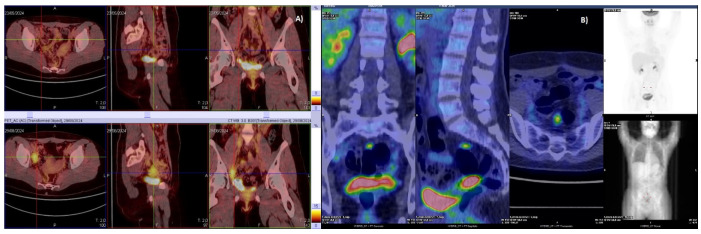
Representative pre-operative functional imaging during recurrent disease. (**A**) Positron emission tomography/computed tomography (PET/CT), 29 August 2024, showing a hypermetabolic pelvic/right iliac lesion suspicious for recurrence. (**B**) PET/CT, 4 March 2025, showing persistent focal pelvic hypermetabolism in the same pelvic region.

**Figure 2:**
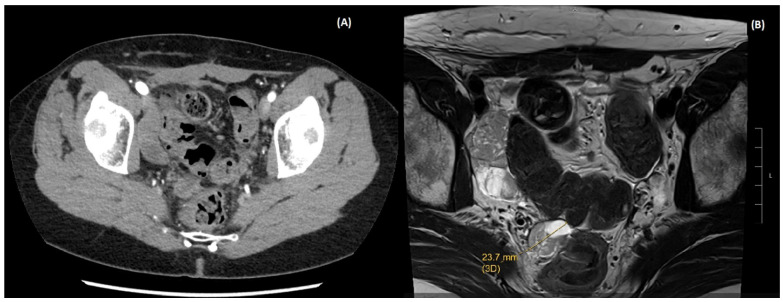
Representative pre-operative cross-sectional imaging before salvage surgery. (**A**) Contrast-enhanced computed tomography (CT), 5 November 2025, showing recurrent pelvic soft-tissue disease. (**B**) Magnetic resonance imaging (MRI), 20 November 2025, showing persistent recurrent pelvic lesions; on the measured image, one lesion is approximately 23.7 mm in greatest dimension.

**Figure 3:**
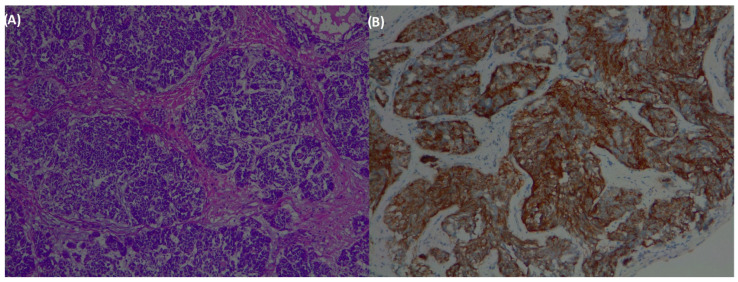
Representative pathology from recurrent disease. (**A**) Hematoxylin and eosin (H&E), 40×, showing a nested and tubule-like neoplastic proliferation within fibrous stroma. (**B**) Cluster of differentiation 117 (CD117)/KIT proto-oncogene receptor tyrosine kinase (c-KIT) immunohistochemistry, 40×, showing membranous and cytoplasmic tumor-cell positivity in the recurrent specimen.

**Table 1:** Condensed diagnostic evolution leading to the final STK11-altered classification.

Diagnostic Phase	Working Diagnosis	Revising Findings	Final Interpretation
Initial 2021 diagnosis	Adult granulosa cell tumor	FOXL2 negativity and STK11 alteration argued against a canonical granulosa-cell neoplasm	Later integrated review favored an STK11-altered adnexal tumor
External 2021 review	High-grade endometrioid carcinoma	Negative staging and later nested/tubule-like recurrence with STK11 alteration weakened a Mullerian carcinoma interpretation	Conventional epithelial ovarian carcinoma no longer fit the cumulative findings
2023 recurrence	Peritoneal metastatic carcinoma	Metastatic behavior was confirmed, but lineage remained unresolved	Serial minimally invasive tissue acquisition enabled later reclassification
Integrated 2024–2025 review	Rare adnexal tumor reconsidered with molecular support	STK11 alteration, FOXL2 negativity, and expert review provided the decisive discordant evidence	Best fit: STK11-altered adnexal tumor of probable mesonephric-remnant origin

**Table 2:** Concise chronology of disease course, diagnostic reassessment, and treatment.

Date	Key Step	Core Finding	Clinical Implication
October 2021	Laparoscopic right salpingectomy	Initial diagnosis: adult granulosa cell tumor	Primary minimally invasive resection
November 2021	External slide review	Reclassified as ovarian endometrioid carcinoma, grade 3	Diagnostic shift
February 2022	Completion surgical staging	No tumor in lymphadenectomy specimens	No disseminated disease documented
January–March 2023	PET/CT plus exploratory laparoscopy	Suspicious right common iliac node; peritoneal metastatic carcinoma	Recurrence confirmed by minimally invasive tissue sampling
May–July 2023	Cisplatin q21d × 4 cycles	Empiric platinum therapy under carcinoma diagnosis	Systemic treatment initiated
October 2023	Exploratory laparoscopy plus pelvic lymphadenectomy	No tumor identified	Suspected nodal relapse not confirmed
October 2024	Cytoreductive surgery	Multifocal abdominal recurrence; postoperative morbidity	Major debulking and completion of adnexal removal
2024–January 2025	Integrated pathology and molecular review	STK11 p.(Leu290Pro), c.869T>C (exon 7) by NGS plus FOXL2 negativity favored STK11-altered adnexal tumor	Histomolecular reclassification
April–May 2025	External review plus everolimus and anastrozole	ER+/PR+ in primary and recurrent specimens; everolimus plus anastrozole adopted	Therapy redirected
September–November 2025	CT/MRI reassessment	Persistent pelvic recurrence and nodal disease	Salvage surgery planned
December 2025	Xipho-pubic laparotomy plus reconstruction	Multifocal pelvic-peritoneal recurrence; CD117 positive in ~40% of cells	Open salvage surgery
April 2026	Early postoperative follow-up	No documented death; patient under surveillance	Outcome data remain immature

**Table 3:** Selected key reports relevant to FATWO-like and STK11-altered adnexal tumors.

Reference	Study Type	Key Contribution	Relevance to the Present Case
Mirkovic et al., 2019 [5]	Genomic profiling	Identified recurrent STK11 alterations in FATWO	Molecular rationale for later reclassification
Bennett et al., 2020 [4]	Clinicopathologic series	Integrated morphology, immunohistochemistry, and molecular data across 15 cases	Refines the differential diagnosis within the Wolffian spectrum
Bennett et al., 2021 [7]	Large case series	Defined a distinctive STK11-altered adnexal tumor, often paratubal and sometimes linked to Peutz-Jeghers syndrome	Strongest support for the final classification adopted here
Estevez-Diz et al., 2021 [8]	Case report	Reported everolimus plus anastrozole in STK11-mutated FATWO	Main therapeutic precedent for the regimen used here
Syriac et al., 2011 [11]	Recurrent-case report	Explored c-KIT gene analysis and possible targeted therapy	Context for interpreting CD117/KIT positivity
Wakayama et al., 2017 [13]	Recurrent-case report	Multimodal salvage with surgery, imatinib, and chemotherapy	Shows the empirical, multimodal nature of recurrent-disease management

## Data Availability

Data supporting the findings of this case report are contained within the article. Additional data are not publicly available because of patient privacy considerations.

## Abbreviations

The following abbreviations are used in this manuscript

FATWO	Female adnexal tumor of probable Wolffian origin
STK11	serine/threonine kinase 11
LKB1	liver kinase B1
mTOR	mammalian target of rapamycin
c-KIT	KIT proto-oncogene receptor tyrosine kinase
CD117	cluster of differentiation 117
PET/CT	positron emission tomography/computed tomography
FOXL2	forkhead box L2
MRI	magnetic resonance imaging
